# Electrochemical hydrodechlorination of perchloroethylene in groundwater on a Ni-doped graphene composite cathode driven by a microbial fuel cell†

**DOI:** 10.1039/c8ra06951d

**Published:** 2018-10-24

**Authors:** Lu Liu, Xiaochen Sun, Wenxin Li, Yonglei An, Hongdong Li

**Affiliations:** State Key Laboratory of Superhard Materials, Jilin University Changchun 130012 China; Key Laboratory of Groundwater Resources and Environment (Jilin University), Ministry of Education Changchun 130021 China anyonglei85@163.com

## Abstract

Enhancing the activity of the cathode and reducing the voltage for electrochemical hydrodechlorination of chlorohydrocarbon were always the challenges in the area of electrochemical remediation. In this study, a novel cathode material of Ni-doped graphene generated by Ni nanoparticles dispersed evenly on graphene was prepared to electrochemically dechlorinate PCE in groundwater. The reduction potential of Ni-doped graphene for PCE electrochemical hydrodechlorination was −0.24 V (*vs.* Ag/AgCl) determined by cyclic voltammetry. A single MFC with a voltage of 0.389–0.460 V and a current of 0.221–0.257 mA could drive electrochemical hydrodechlorination of PCE effectively with Ni-doped graphene as the cathode catalyst, and the removal rate of PCE was significantly higher than that with single Ni or graphene as the cathode catalyst. Moreover, neutral conditions were more suitable for Ni-doped graphene to electrochemically hydrodechlorinate PCE in groundwater and no byproduct was accumulated.

## Abstract 2

Ni-doped graphene was prepared to electrochemically dechlorinate PCE driven by a microbial fuel cell. Dechlorination efficiency and reduction potential were significantly higher than for bare Ni or graphene.

## Graphical abstract

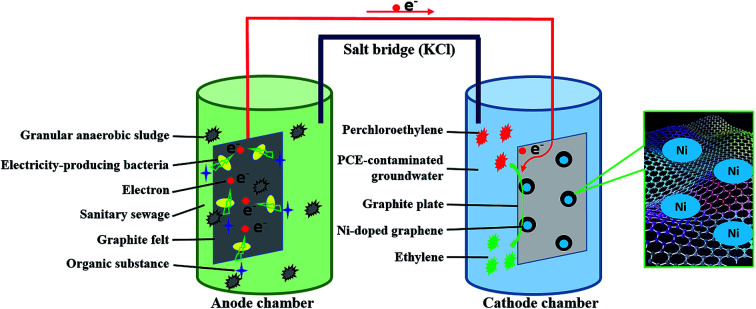

## 1. Introduction

Perchloroethylene (PCE) is used widely as an organic solvent and degreasing agent.^1,2^ PCE is also a typical refractory contaminant in groundwater due to its improper handling and disposal practices.^3,4^ Hydrodechlorination is an efficient way to eliminate PCE contamination because PCE can be dechlorinated into less chlorinated ethylene such as cDCE (*cis*-dichloroethylene), VC (vinyl chloride) or ETH (ethylene).^5–7^ Hydrodechlorination mainly involves microbial hydrodechlorination,^8–10^ chemical hydrodechlorination^11,12^ and electrochemical hydrodechlorination.^13,14^

Microbial hydrodechlorination usually occurs with dechlorinating bacteria under strict anaerobic conditions.^8^ Although microbial hydrodechlorination exhibits excellent decontamination of PCE, three unsolved problems hinder the application of microbial hydrodechlorination for PCE-contaminated groundwater remediation. First, the difficulty of controlling anaerobic dechlorinating bacteria limits the reliability of microbial hydrodechlorination because the ability to completely dechlorinate PCE seems to be restricted to microorganisms belonging to the genus *Dehalococcoides*.^15–17^ Second, fierce competition for a carbon source and hydrogen between dechlorinating bacteria and other bacteria (such as sulfate-reducing bacteria and methanogenic bacteria) will decrease the dechlorination effects.^16^ Third, a chemical electron donor (*e.g.* acetate) is necessary for dechlorinating bacteria, and the accumulation of microbial fermentation products and microorganisms can cause groundwater clogging.^15,18^

Chemical hydrodechlorination generally uses chemical reductant (*e.g.* Zero Valent Iron, ZVI) as electron donor for PCE dechlorination.^19^ To enhance the hydrodechlorination efficiency, bimetals (*e.g.* Fe/Ni) are widely used for hydrodechlorination of chlorinated aliphatic hydrocarbons.^20^ In our previous study, we found that bimetals nano-Fe/Ni was more effective than single nano-Fe for PCE hydrodechlorination in groundwater. However, both nano-Fe and nano-Ni tend to aggregate due to their high interface energy and inherent magnetism, which can significantly decrease the hydrodechlorination efficiency.^20^ In addition, nano-Fe and nano-Ni will certainly cause groundwater contaminated by heavy metals according to the reactions: Fe − 2e^−^ → Fe^2+^, Ni − 2e^−^ → Ni^2+^.

Electrochemical hydrodechlorination directly utilizes external power as the electron donor, chlorinated aliphatic hydrocarbons (*e.g.* PCE) can be dechlorinated on the catalytic cathode through obtaining electrons and protons.^21,22^ Compared to microbial hydrodechlorination and chemical hydrodechlorination, electrochemical hydrodechlorination needs not to cultivate dechlorinating bacteria and inject chemical electron donors into groundwater. Hence, electrochemical hydrodechlorination is recognized as an efficient technology with potential and development for chlorohydrocarbon-contaminated groundwater remediation. However, electrochemical hydrodechlorination usually is driven by external power with the voltages varied from 5–20 V, which may cause undesired reactions (*e.g.* water electrolysis) and consume plenty of electric energy.^18,23^ In addition, cathode material has great influences on the results of electrochemical hydrodechlorination.^24^ Catalytic cathode can combine electrons and protons (or water moleculars) to generate activated hydrogen atoms which subsequently act on chlorohydrocarbon (*e.g.* PCE) so as to accomplish hydrodechlorination process.^25^

Currently, carbon materials (*e.g.* particle graphite) and metal materials (*e.g.* Pt, Pd, Ni, Cu, Zn, Ag, Pb, stainless steel.) are widely used as cathode materials.^26–29^ Especially noble metals such as Pt and Pd have excellent catalytic property for electrochemical hydrodechlorination due to their low electric potential of producing hydrogen and high capacity of adsorbing hydrogen.^30–32^ However, high cost actually limits the application of noble metals Pt and Pd.^33^ Fortunately, it had been verified that metal Ni also has the catalytic property for electrochemical hydrodechlorination, although the catalytic activity is lower than Pt and Pd.^34–36^ Therefore, some efforts such as decreasing dechlorinating voltage, saving electric energy and enhancing the catalytic activity of Ni-cathode, should be made to improve the electrochemical hydrodechlorination of chlorohydrocarbon. Recent years, graphene is widely used as catalyst carrier due to its excellent characteristics such as low resistivity, high thermal conductivity and mechanical strength.^37–39^ When metal nanoparticles load on the surface of graphene, the catalytic activity can be enhanced significantly, and agglomeration of magnetic nanoparticles (*e.g.* nano-Ni) can be decreased effectively.^40,41^ However, electrochemical hydrodechlorination of PCE in groundwater on Ni-doped graphene cathode has no report so far.

Microbial fuel cell (MFC) has been widely studied in the area of anaerobic biodegradation of organic pollutants in recent years, which can not only eliminate organic pollution with anaerobic electrogenesis microorganisms, but also produce electric energy through electronic transportation in external circuit. However, it is a pity that the open circuit voltage of single MFC is so small (the maximum is about 0.7 V) that the electric energy is hard to collect or utilize directly.^42^ Fortunately, it had been reported that PCE/TCE (trichloroethylene) can be electrochemically dechlorinated under high cathode potentials varied from −450 to −550 mV (*vs.* SHE).^16,17^ Hennebel also found that TCE can be effectively dechlorinated under the voltage of 0.8 V in microbial electrolysis cells with biogenic palladium nanoparticles.^23^ These demonstrated that it is feasible to utilize MFC to electrochemically dechlorinate PCE as long as the hydrodechlorinating catalyst on cathode is proper. However, electrochemical hydrodechlorination of PCE in groundwater with MFC has not been reported yet.

This study aimed to prepare Ni-doped graphene as the cathode material and use MFC (based on anaerobic sanitary sewage treatment) as the electric power to electrochemically dechlorinate PCE in groundwater. This study would develop an efficient cathode material for electrochemical hydrodechlorination and exhibit a novel remediation technology for PCE-contaminated groundwater.

## 2. Materials and methods

### 2.1 Preparation of Ni-doped graphene

Ni (nickel formate dihydrate, A.R.) and graphene (*D*_50_ : 7–12 μm, monolayer content >80%) were dispersed sufficiently in absolute ethanol according to stoichiometric ratio 1 : 10. Then, the solid mixture of Ni and graphene heated in tube furnace under 400 °C for 2 hours with nitrogen flow after ethanol evaporated completely under room temperature.

### 2.2 Characterization of Ni-doped graphene

The microstructure of Ni-doped graphene was indicated by transmission electron microscope (JEM-2200FS JEOL Japan). The crystal lattice structures of Ni-doped graphene were showed by XRD (D/max-2550 RigaKu Japan) and HRTEM (JEM-2200FS JEOL Japan).

### 2.3 Electrochemical measurements

Electrochemical performance of Ni-doped graphene electrode was determined using a CHI660E electrochemical workstation (Shanghai Chenhua Instrument Co. Ltd., Shanghai, China). Three electrode systems consisting of Ni-doped graphene electrode, Ag/AgCl electrode, and Pt wire as working, reference, and counting electrodes, respectively, were used. The electrochemical analysis was performed with cyclic voltammetry in a 1 mM PCE solution containing 0.1 M KCl. All analytical measurements were performed at room temperature.

### 2.4 PCE-contaminated groundwater

PCE-contaminated groundwater was prepared in laboratory with the actual uncontaminated groundwater which was taken from a groundwater well of Changchun City. The hydrochemical components of groundwater contained 661.94 mg L^−1^ of salinity, 0.76 mg L^−1^ of K^+^, 65.53 mg L^−1^ of Na^+^, 162.27 mg L^−1^ of Ca^2+^, 231.51 mg L^−1^ of HCO_3_^−^, 193.08 mg L^−1^ of Cl^−^, 122.25 mg L^−1^ of SO_4_^2−^, 1.30 mg L^−1^ of NO_3_^−^, 0.01 mg L^−1^ of NO_2_^−^, 0.46 mg L^−1^ of F^−^, 7.05 of pH. Before adding PCE, the raw groundwater was put into anaerobic glove box (COY, USA) until dissolved oxygen (DO) was not detected by DO meter (310D-01A, ORION).

### 2.5 Set-up of MFC

Two-chambered MFC was constructed with anaerobic microbial anode and air-aerated cathode. Anaerobic activated sludge was taken from a sewage treatment plant of Changchun City. Anolyte was the raw groundwater dissolved with beef extract, which was simulated as sanitary sewage. The substrate of anode was a piece of graphite felt (100 mm × 50 mm × 2 mm). Salt bridge composed of agar and saturated KCl was used to connect anolyte and catholyte. Anode and cathode were connected by copper wires with a battery (Nanfu, 1.5 V) which was used to induce anaerobic electrogenesis microorganisms to preferentially inhabit on the graphite felt fastly. The battery would be dismantled as soon as the open circuit voltage and electric current of MFC maintained above 0.45 V and 0.25 mA, respectively.

### 2.6 Remediation of PCE-contaminated groundwater

**Fig. 1:**
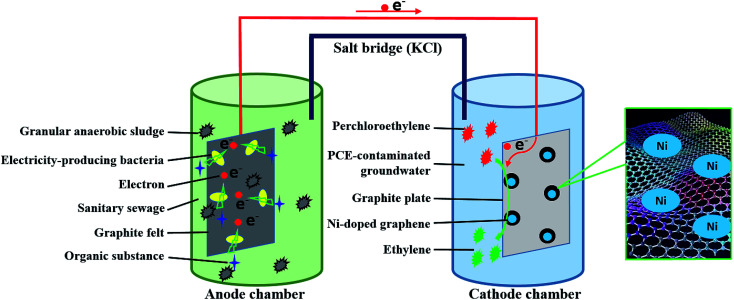
Experimental schematic diagram of this study.

The cathode of MFC was changed into Ni-doped graphene cathode instead of air-aerated cathode. The catholyte was PCE-contaminated groundwater which was sealed with rubber stopper to guard against oxygen intrusion and PCE volatilization. Ni-doped graphene cathode was prepared with Ni-doped graphene powder and graphite plate (50 mm × 50 mm × 3 mm). The Ni-doped graphene powder loaded on one side of graphite plate (50 mm × 50 mm) through heating the graphite plate on which the solution containing 11 mg Ni-doped graphene (10 mg graphene and 1 mg Ni) dispersed in ethanol was coated evenly in tube furnace under 400 °C for 2 hours with nitrogen flow. During the experimental process, concentration of PCE and corresponding degradation products such as TCE, cDCE, VC and ETH, were determined by Gas Chromatography-Flame Ionization Detector (GC 2010, Shimadzu). The open circuit voltage and electric current of MFC were also monitored periodically by an accurate multimeter (VC890D, Victor). The experimental schematic was described in Fig. 1.

### 2.7 Calculation methods

The calculation formula of dechlorination efficiency for PCE was described as follow:1
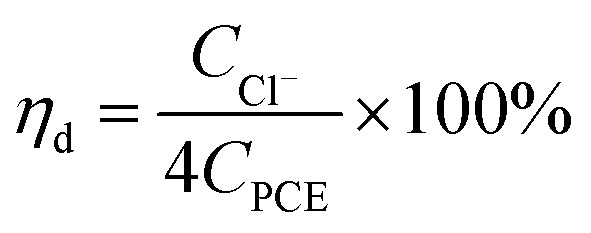
where, *η*_d_ is the dechlorination efficiency, unit: %; *C*_Cl^−^_ is the concentration of chloridion, unit: mmol L^−1^; *C*_PCE_ is the initial concentration of PCE, unit: mmol L^−1^; the number 4 represents one PCE corresponding to four chloridion.

The calculation formula of coulombic efficiency for microbial anode was described as follow:2
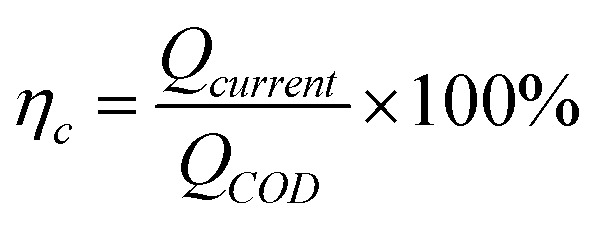

3 *Q*_current_ = *I*δ*t*

4
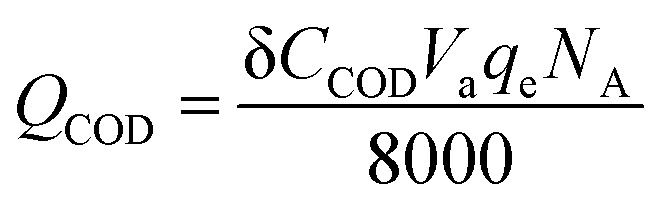
where, *η*_c_ is the coulombic efficiency, unit: %; *Q*_current_ is the electric quantity of external circuit, unit: C; *Q*_COD_ is the theoretical electric quantity produced by microbial electrogenesis of anolyte COD, unit: C; *I* is the current of external circuit, unit: A; δ*t* is the time frame to calculate coulombic efficiency, unit: s; δ*C*_COD_ is the concentration difference of anolyte COD, unit: mg L^−1^; *V*_a_ is the volume of anolyte, unit: L; *q*_e_ is electronic charge, 1.6 × 10^−19^ C; *N*_A_ is Avogadro constant, 6.02 × 10^23^ mol^−1^.

The calculation formula of current efficiency for Ni-doped graphene cathode can be described as follow:5
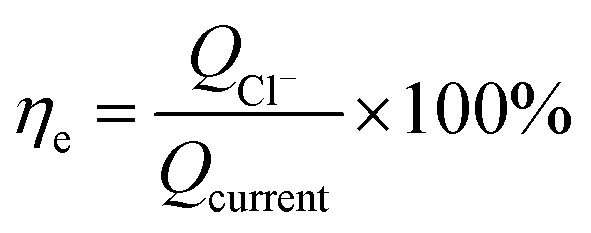
6
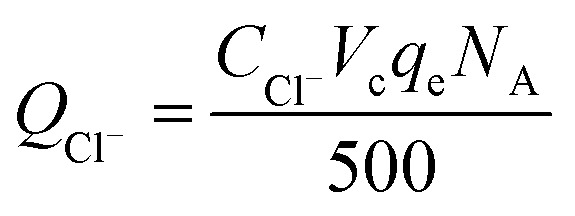
where, *η*_e_ is the current efficiency, unit: %; *Q*_Cl^−^_ is the total electric quantity used for electrochemical hydrodechlorination of PCE, unit: C; *Q*_current_ is same as formula (3); *C*_Cl^−^_ is the concentration of chloridion, unit: mmol L^−1^; *V*_c_ is the volume of catholyte, unit: L; *q*_e_ is electronic charge, 1.6 × 10^−19^ C; *N*_A_ is Avogadro constant, 6.02 × 10^23^ mol^−1^.

## 3. Results and discussion

### 3.1 Characterization of Ni-doped graphene

**Fig. 2:**
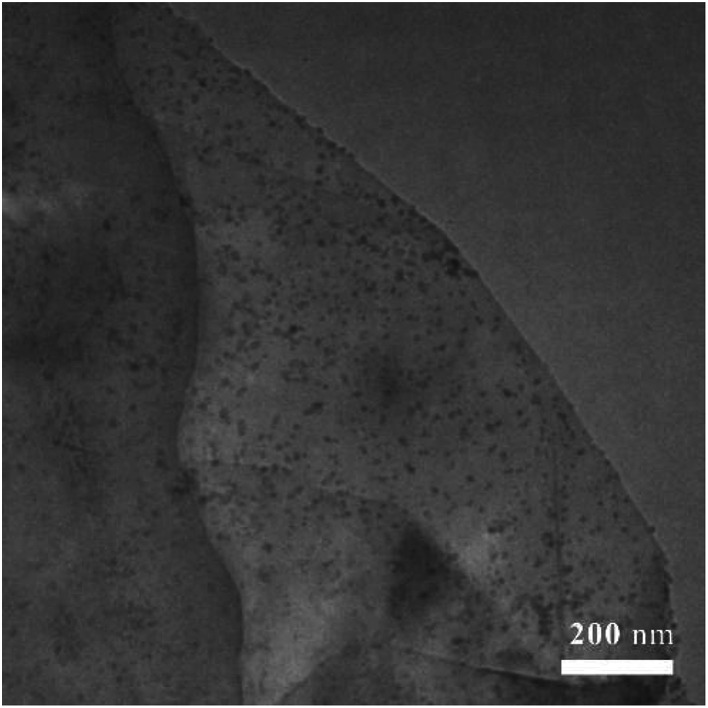
Transmission electron microscopic (TEM) image of Ni-doped graphene.

Transmission electron micrographies of Ni-doped graphene had been recorded using a copper grid dipped in a solution containing Ni-doped graphene particles dispersed in ethanol by ultrasonication and presented in Fig. 2. TEM photo revealed the presence of a large number of nickel particles with uniform size and well dispersed on the graphene.

**Fig. 3:**
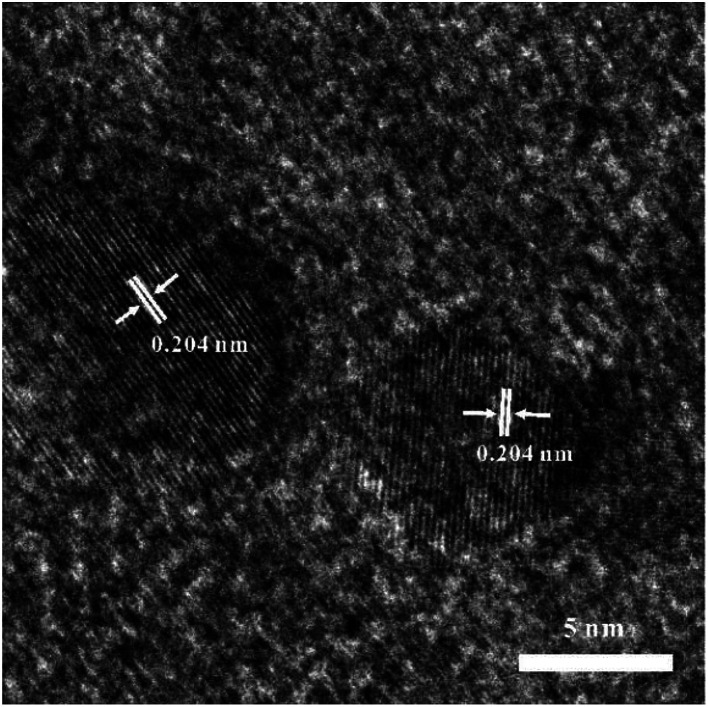
High resolution transmission electron microscopic (HRTEM) image of Ni-doped graphene.

A high-resolution transmission electron microscopic (HRTEM) image was given in Fig. 3, where most particles had sizes of about 5–10 nm, and the lattice fringe spacing was 0.204 nm, corresponding to (111) crystal planes of cubic nickel (JCDPS# 04-0850).

**Fig. 4:**
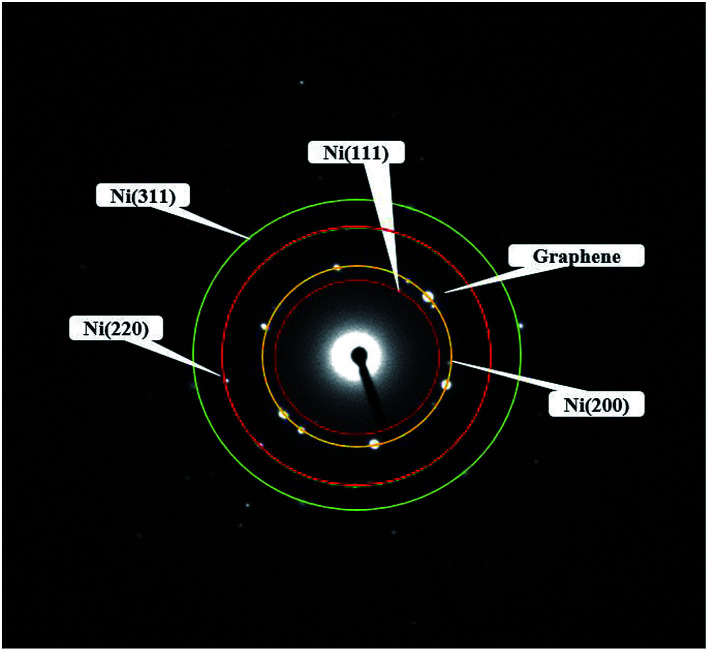
Electron diffraction pattern of Ni-doped graphene.

Fig. 4 showed the selected area electron diffraction (SAED) pattern of the Ni-doped graphene. The appearance of strong diffraction spots rather than diffraction rings confirmed the formation of single crystalline cubic nickel. The ratio of the square of the ring radius was 3 : 4 : 8 : 11, which indicated that the structure was cubic nickel type, and the rings corresponded to the (111), (200), (220), and (311) crystal planes of cubic nickel structure.^43,44^

**Fig. 5:**
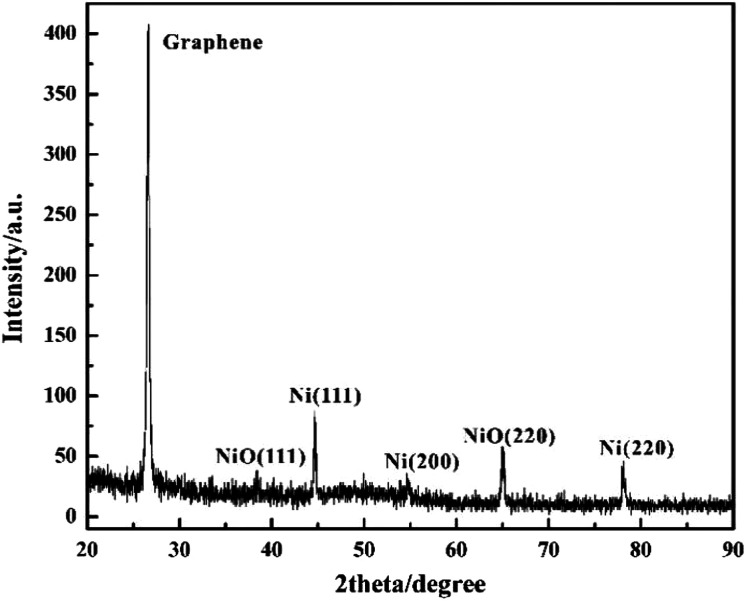
XRD spectra of Ni-doped graphene.

The phase and crystallinity of Ni-doped graphene were characterized by using a Rigaku X-ray diffractometer with Cu K radiation over a range of 2*θ* angles from 20° to 90° (Fig. 5). A sharp and strong typical peak corresponding to graphene appeared at the 2*θ* angle of 26.6°.^45^ Simultaneously, the peaks located at the 2*θ* angles of 44.7°, 54.6° and 78.0°, indicated the (111), (200) and (220) crystal planes of cubic nickel lattice, respectively.^43,44^ These results confirmed that Ni nanoparticles had been dispersed on the graphene evently. However, the peaks corresponding to (111) and (220) crystal planes of nickel oxide lattice appeared at the 2*θ* angles of 38.4° and 65.0°, which indicated that some nickel oxide was produced due to the exposure of Ni-doped graphene to air.^46,47^

### 3.2 Cyclic voltammetry behavior of Ni-doped graphene

**Fig. 6:**
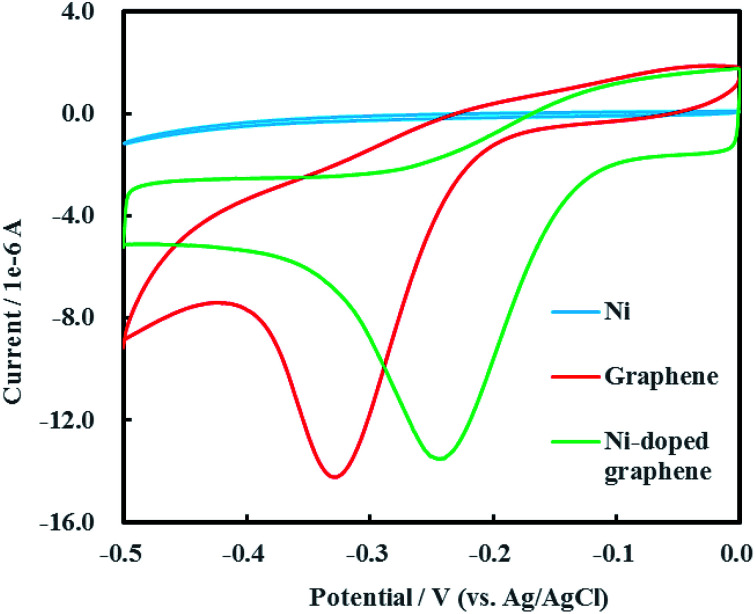
Cyclic voltammogram of Ni-doped graphene.

Cyclic voltammogram of Ni-doped graphene was shown in Fig. 6 after multiple electrochemical scans. A sharp and strong reductive peak located at −0.24 V (*vs.* Ag/AgCl) was observed in the cyclic voltammogram of Ni-doped graphene. The cyclic voltammograms of single Ni and graphene were also conducted as the experimental controls. It was found that the reductive peak of graphene was located at −0.33 V (*vs.* Ag/AgCl) which was significantly lower than the reduction potential of Ni-doped graphene. An interesting result was shown that single Ni had no reductive peak over a range of potential from −0.50 to 0 V (*vs.* Ag/AgCl). These results demonstrated that the Ni-doped graphene can be used as catalytic composited cathode material for electrochemical hydrodechlorination of PCE under low voltage (0.24 V) which was significantly lower than those reported up to now.^16–18,23^

### 3.3 Remediation efficiency of PCE-contaminated groundwater

**Fig. 7:**
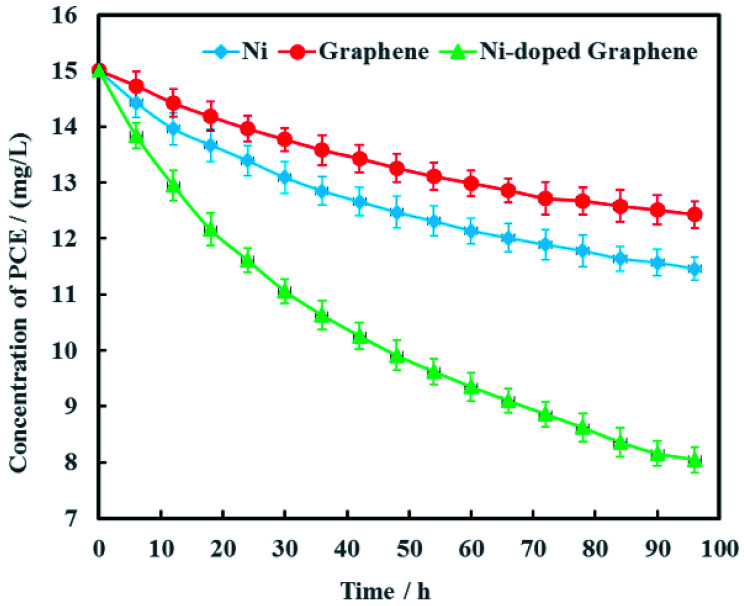
Removal efficiency of PCE with Ni-doped graphene cathode (error bars represent the standard deviation of triplicate runs).

Electrochemical hydrodechlorination of PCE in groundwater driven by MFC was carried out on the graphite cathode coated with Ni-doped graphene. As the experimental controls, single Ni and graphene were also used as cathode materials to electrochemically dechlorinate PCE in groundwater, respectively (Fig. 7). The results showed that PCE can be removed effectively with Ni-doped graphene although PCE also can be electrochemically removed with single Ni or graphene. At the remediation time of 96 h, the removal rates of PCE were 23.6%, 17.1% and 46.3% with the cathode materials Ni, graphene and Ni-doped graphene, respectively (Fig. 7). These results demonstrated that the hydrodechlorination activity of Ni-doped graphene was actually higher than single Ni or graphene, resulting from the synergistic effect between superconductivity of graphene and high surface catalytic activity of nano-Ni particles.

**Fig. 8:**
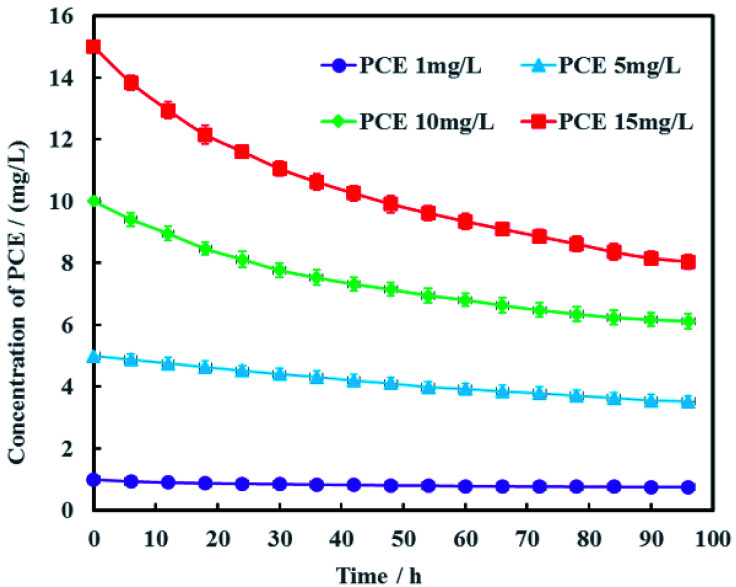
Effects of PCE concentration on electrochemical hydrodechlorination efficiency (error bars represent the standard deviation of triplicate runs).

The concentration of PCE in actual groundwater are varied along the contamination plume. Therefore, the effects of PCE concentration on electrochemical hydrodechlorination efficiency was investigated in this study and the results were shown in Fig. 8. It was obvious that the higher the initial PCE concentration was, the higher PCE removal rate was. At the remediation time of 96 h, the removal rates of PCE were 24.5%, 29.4%, 38.8% and 46.3% with the different initial PCE concentrations of 1, 5, 10 and 15 mg L^−1^, respectively. These results suggested that the electrochemical hydrodechlorination efficiency of PCE had positive correlation with PCE concentration in groundwater. For low concentration of PCE, it would be spent more time to eliminate PCE contamination completely in groundwater. In addition, the total amount of nickel in Ni-doped graphene cathode was only 1 mg, which may lead to low removal rate of PCE. Therefore, the amount of Ni-doped graphene coated on cathode would be optimized in future study.

**Fig. 9:**
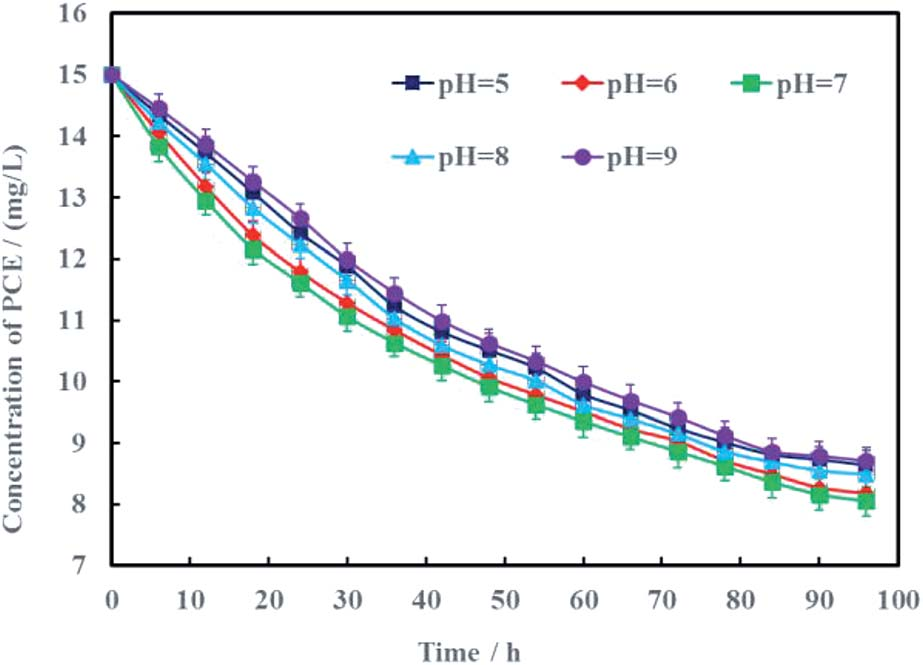
Effects of pH on electrochemical hydrodechlorination efficiency of PCE (error bars represent the standard deviation of triplicate runs).

Proton is an important reactant for electrochemical hydrodechlorination of PCE. According to the hydrodechlorination mechanism of PCE, protons combined with electrons would replace the chlorines of PCE.^25^ Generally speaking, the pH of groundwater often varies from 5 to 9. Therefore, effects of pH on electrochemical hydrodechlorination efficiency of PCE in groundwater was investigated in this study and the results were shown in Fig. 9. It can be seen clearly that pH had nonsignificant effect on PCE hydrodechlorination (*p* < 0.05). At the remediation time of 96 h, the removal rates of PCE were 41.4%, 43.5%, 46.3%, 42.1% and 40.3% under different initial pH of 5, 6, 7, 8 and 9, respectively. These results demonstrated that neutral condition was more suitable for Ni-doped graphene to electrochemically hydrodechlorinate PCE. The cathode material of Ni-doped graphene prepared in this study can be better used for electrochemical hydrodechlorination remediation of actual PCE-contaminated groundwater.

### 3.4 Electrochemical hydrochlorination mechanism

To electrochemically dechlorinate PCE in groundwater with aboveground MFC as the electric driver, salt bridge had to be utilized to connect the microbial anode chamber and PCE-contaminated groundwater cathode chamber. However, the resisitance of entire electrochemical remediation system was so high (Fig. S2, ESI†) that the loop current was fairly low (Fig. 12), which actually led to low dechlorination efficiency of PCE. Therefore, more time was spent for PCE electrochemical hydrodechlorination and the degradation products such as TCE, cDCE, VC, ETH and chloridion were monitored synchronously.

**Fig. 10:**
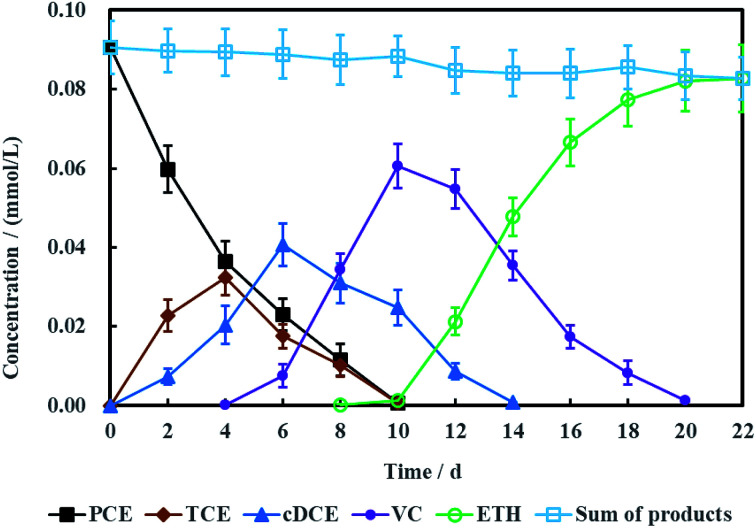
Performance of PCE electrochemical hydrodechlorination with Ni-doped graphene cathode (error bars represent the standard deviation of triplicate runs).

Fig. 10 showed the concentration variations of PCE and corresponding dechlorination products. PCE can be removed completely in 10 days, companied with the appearance and disappearance of TCE. cDCE and VC also were detected in the electrochemical reduction system. The maximum concentration of cDCE was detected at 6 days and completely eliminated at 14 days. VC appeared at 4 days and completely disappeared at 20 days. Finally, ETH was the only product for PCE electrochemical hydrodechlorination. Generally, the process of PCE electrochemical hydrodechlorination can be described as follow:^48^

7 C_2_Cl_4_ + *m*e^−^ + *n*H^+^ → C_2_H_*n*_Cl_4−*m*+*n*_ + (*m* − *n*)Cl^−^

Therefore, TCE, cDCE, VC and ETH were the common dechlorination products for PCE.

**Fig. 11:**
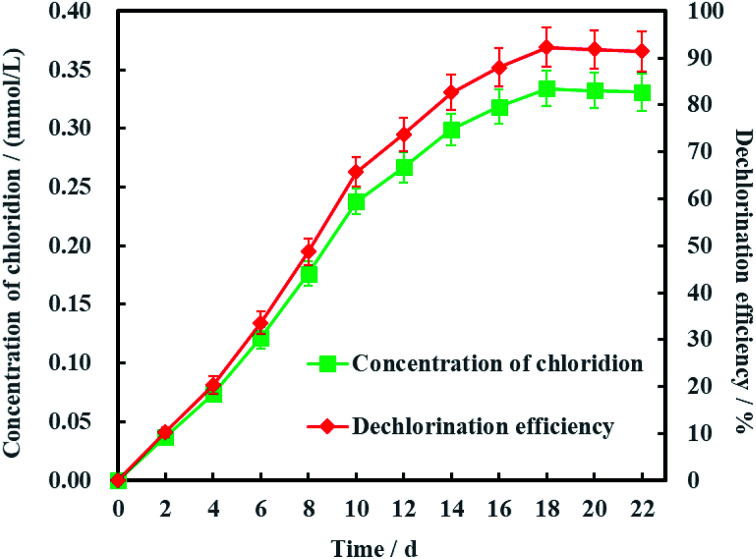
Dechlorination efficiency calculated by the concentration of chloridion (error bars represent the standard deviation of triplicate runs).

As is known to all, less chlorinated ethylenes (*e.g.* TCE, cDCE and VC) is more difficult to be dechlorinated than PCE due to asymmetric p–π conjugation effects. Hence, the dechlorination process from cDCE and VC to ETH usually is the limited step for dechlorinating PCE into ETH.^18^ Therefore, Ni-doped graphene most probably can be better used for electrochemical hydrodechlorination of less chlorinated ethylenes. In addition, the detection of chloridion (Fig. 11) and uncorrosion of nickel (Fig. S1, ESI†) further certified the electrochemical hydrodechlorination of PCE, and the dechlorination efficiency of PCE calculated by the concentration of chloridion was 91.40% at 22 days.

### 3.5 Electrical characteristics of MFC

**Fig. 12:**
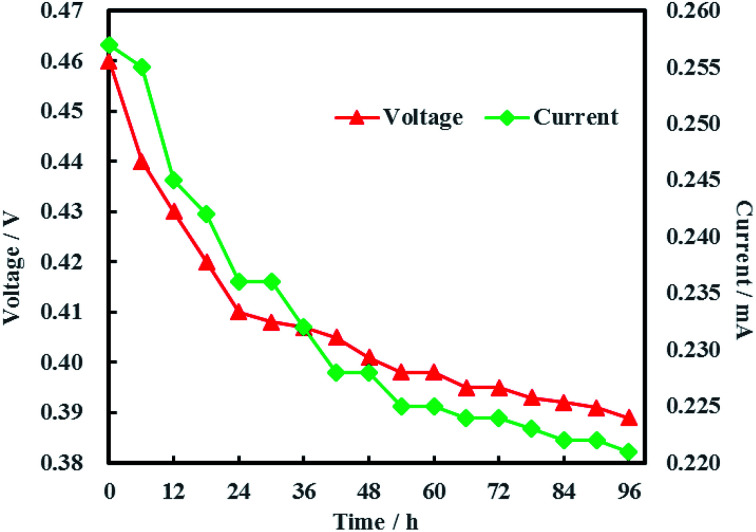
Open circuit voltage and current of MFC.

**Fig. 13:**
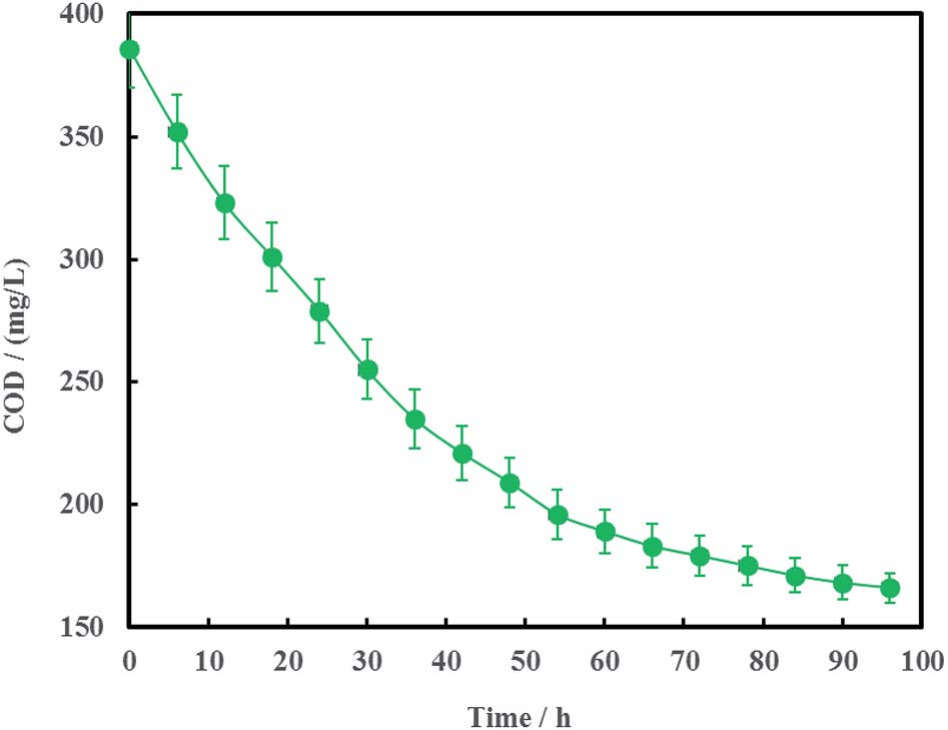
Variation of anolyte COD (error bars represent the standard deviation of triplicate runs).

The open circuit voltage and current were monitored regularly to investigate the electrical characteristics of MFC. The results in Fig. 12 showed that both the open circuit voltage and current decreased gradually from 0.460 to 0.389 V and 0.257 to 0.221 mA during the whole 96 h operation process. The variation of anolyte COD was also investigated in this study and the results showed that anolyte COD also decreased gradually from 386 to 166 mg L^−1^ (Fig. 13). Thus it can be inferred that the decreasements of open circuit voltage and current were caused by reduction of anolyte COD. Fortunately, the open circuit voltage of MFC was always more than 0.24 V consistently, which meant that electrochemical hydrodechlorination of PCE could occur from beginning to end with Ni-doped graphene cathode.

**Fig. 14:**
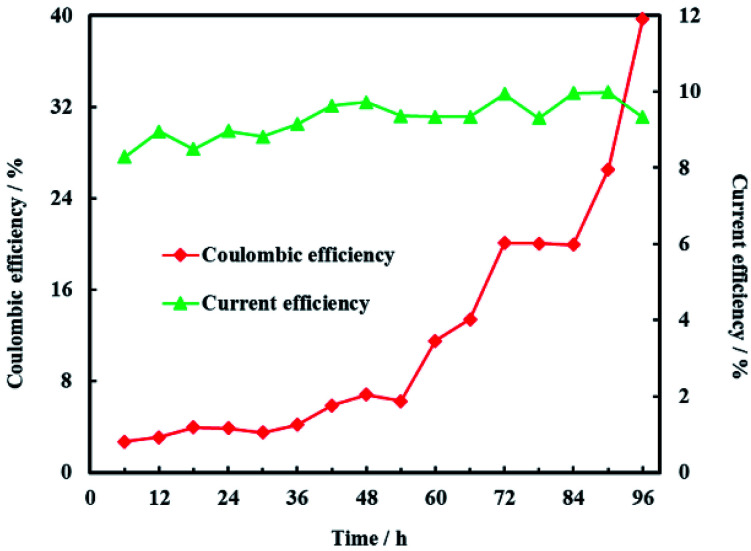
Coulombic efficiency of anode and current efficiency of cathode.

Coulombic efficiency of MFC is used to assess the transfer efficiency of microbial fuel in anolyte from chemical energy to electric energy. It can be seen from Fig. 14 that coulombic efficiency increased gradually from 2.70% to 39.74% and the increasing trend after 54 h was significantly faster than that before 54 h. The average coulombic efficiency was 11.98%. During the remediation time of 0–54 h, open circuit voltage, current and anolyte COD also fastly decreased to 0.398 V, 0.225 mA and 196 mg L^−1^, respectively. These results suggested that rapid degradation of anolyte COD with high concentration (>196 mg L^−1^) didn't mean that more electric energy would be gained from MFC. On the contrary, low concentration (<196 mg L^−1^) of anolyte COD would enhance the coulombic efficiency of MFC although the total electric energy was relative less. The reason for increasing coulombic efficiency most probably was that electrons produced by microorganisms were partially absorbed by the electron acceptors (such as O_2_, NO_3_^−^, *etc.*) in anolyte before 54 h, rather than flowed into cathode *via* external circuit. After all, it is easy for O_2_ and NO_3_^−^ to trap electron and react as follow:^17^

8 O_2_ + 4H^+^ + 4e^−^ → 2H_2_O, *E*^0^ = 1.23 V *vs.* SHE

9 NO_3_^−^ + 3H_2_O + 5e^−^ → 1/2N_2_ + 6OH^−^, *E*^0^ = 0.26 V *vs.* SHE

In addition, microorganisms would consume more COD when O_2_ and NO_3_^−^ rather than electrode were the microbial electron acceptors, which directly led to low coulombic efficiency before 54 h. Therefore, the anolyte of MFC should be optimized before application for electrochemical hydrodechlorination of PCE so as to save microbial fuel and raise the electric energy yield.

Current efficiency is used to assess the utilization efficiency of current produced by MFC on cathode. It was obvious that current efficiency of cathode was steady and the average current efficiency was only 9.28% (Fig. 14). The reason for the low current efficiency most probably was the electrochemical reduction of other electron acceptors such as NO_3_^−^ and SO_4_^2−^ in groundwater.^48^ In addition, production of H_2_ on the Ni-doped graphene could also decreased the current efficiency due to the lower hydrogen evolution overpotential of nickel.^48^ This is why electrochemical hydrodechlorination is hard to completely eliminate PCE in groundwater. Therefore, the effects of multi-electron acceptors on electrochemical hydrodechlorination of PCE would be investigated in future study.

## 4. Conclusions

A novel cathode material of Ni-doped graphene for electrochemical hydrodechlorination of PCE was prepared and investigated successfully in this study. Ni nanoparticles with 5–10 nm size dispersed on the graphene evenly. The reduction potential of Ni-doped graphene for PCE electrochemical hydrodechlorination was −0.24 V (*vs.* Ag/AgCl), which was significantly lower than those reported up to now. Electrochemical hydrodechlorination of PCE with Ni-doped graphene could be driven by low-voltage MFC, and the hydrodechlorination efficiency of PCE with Ni-doped graphene as the cathode material was obviously higher than that with single Ni or graphene. Most important was that Ni-doped graphene had the best PCE removal efficiency under neutral condition and no byproduct was accumulated.

## Conflicts of interest

There are no conflicts to declare.

## Supplementary Material

RA-008-C8RA06951D-s001
