# Supplementary material for: Electrochemical hydrodechlorination of perchloroethylene in groundwater on a Ni-doped graphene composite cathode driven by a microbial fuel cell
Source: RSC Adv. 2018 Oct 24;8(63):36142–9. doi: 10.1039/c8ra06951d (PMC9088688; doi:10.1039/c8ra06951d)
Supplement: RA-008-C8RA06951D-s001 [file RA-008-C8RA06951D-s001.pdf]

Electronic Supplementary Material (ESI) for RSC Advances.
This journal is © The Royal Society of Chemistry 2018

Electrochemical Hydrodechlorination of Perchloroethylene in groundwater on Ni-doped Graphene Composite Cathode Driven by Microbial Fuel Cell

Lu Liu[a], Xiaochen Sun[a], Wenxin Li[b], Yonglei An[b*], Hongdong Li[a]

[a] State Key Laboratory of Superhard Materials, Jilin University, Changchun 130012, China

[b] Key Laboratory of Groundwater Resources and Environment (Jilin University), Ministry of Education, Changchun, 130021, China

[*] Correspondence: anyonglei85@163.com

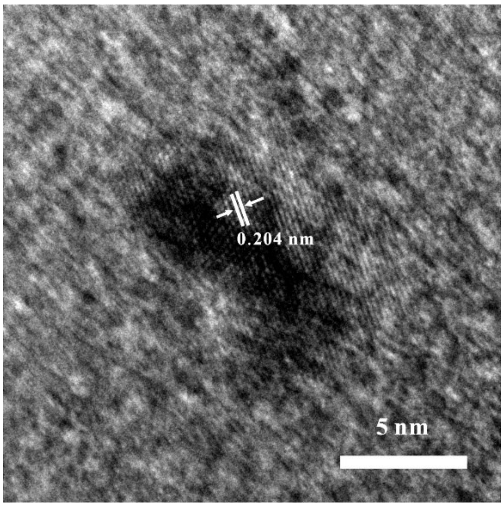

Figure S1 High resolution transmission electron microscopic (HRTEM) image of the Ni-doped graphene after 22-day reaction for electrochemical hydrodechlorination of PCE. The 0.204 nm of lattice fringe spacing corresponding to (111) crystal plane of cubic nickel sufficiently certifed that the nickel in Ni-doped graphene cathode was not corroded during the process of electrochemical hydrodechlorination of PCE.

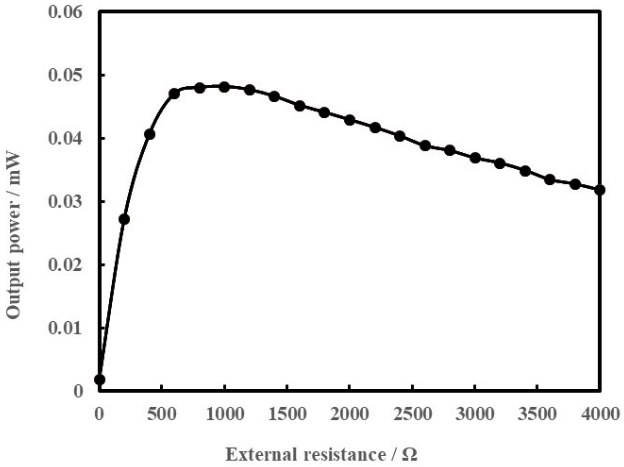

Figure S2 The relationship between external resistance and output power of MFC. As is known to all, the maximum output power will be attained when the internal resistance of battery and the external resistance are equal. Therefore, it can be seen from Figure S2 that the internal resistance of the MFC in this study was about 800 Ω.
